# Obstructive Sleep Apnea and Chronic Rhinosinusitis: Understanding the Impact of OSA on CRS Disease Burden

**DOI:** 10.1002/ohn.934

**Published:** 2024-11-22

**Authors:** Emily Garvey, Alexander Duffy, Sruti Tekumalla, Bita Naimi, Chase Kahn, Angela Yang, Zachary Urdang, Douglas Farquhar, Marc Rosen, Gurston G. Nyquist, Elina Toskala, Mindy Rabinowitz

**Affiliations:** ^1^ Department of Otolaryngology Head and Neck Surgery Thomas Jefferson University Hospital Philadelphia Pennsylvania USA

**Keywords:** chronic rhinosinusitis, obstructive sleep apnea, quality of life

## Abstract

**Objective:**

Assess the impact of obstructive sleep apnea (OSA) on chronic rhinosinusitis (CRS) severeity.

**Study Design:**

Retrospective database review.

**Setting:**

TriNetX US database.

**Methods:**

TriNetX US Collaborative Network database was queried for cohorts of patients with OSA, CRS, and CRS with comorbid OSA (CRS‐OSA). Data included demographics, CRS severity was assessed via rates of endoscopic sinus surgery (ESS), antibiotic, and oral steroid use. Propensity score matching was performed to account for differences in demographics and clinical variables.

**Results:**

The query identified 1,818,879 patients with CRS, 481144 with OSA, and 93,153 CRS‐OSA patients. OSA‐CRS patients had higher rates of hypertension, diabetes mellitus, obesity, and asthma than either CRS or OSA populations (*P* < 0.0001). CRS‐OSA patients demonstrated higher rates of ESS (odds ratio [OR]: 1.91, 1.82‐2.02, *P* < 0.0001), antibiotic (OR: 1.90, 1.81‐1.96, *P* < 0.001), and oral steroid use (OR: 2.23, 2.16‐2.28, *P* < 0.001) compared to CRS‐only patients. CRS‐OSA patients not on continuous positive airway pressure had higher utilization of antibiotics (OR: 3.24, 2.82‐3.71, *P* < 0.0001) and steroids (OR: 2.28, 2.05‐2.55, *P* < 0.0001) than nonutilizers. CRS‐OSA patients with sleep‐related surgical interventions required fewer antibiotic courses (OR: 1.93, 1.62‐2.28, *P* < 0.0001).

**Conclusion:**

CRS‐OSA patients experience higher rates of comorbidities associated with both diseases than those with CRS or OSA alone. OSA was associated with an increased risk of ESS, antibiotic, and steroid use in patients with CRS. There appears to be a correlation with treatment of OSA and CRS outcomes, however, further studies are required.

Chronic rhinosinusitis (CRS) is one of the most common chronic conditions and affected patients report high rates of sleep dysfunction and increased rates of mood disorders.^1^, ^2^ Previous literature has demonstrated a relationship between CRS and obstructive sleep apnea (OSA). In recent reports 10 to even 60% of CRS patients are reported to experience comorbid OSA.^3^ The pathophysiologic connection between CRS and OSA is not fully understood, however recent studies have demonstrated demographic connections between the 2 populations, such as high rates of obesity. Additionally current literature suggests that the chronic intermittent apneic episodes in patients with OSA lead to increased activation in inflammatory pathways leading to production of proinflammatory cytokines including interleukin (IL)‐1, IL‐6, IL‐8, tumor necrosis factor‐α, and C‐reactive protein.^4^, ^5^

Studies have demonstrated that treating nasal obstruction in patients with OSA leads to overall improved quality of life metrics including a decrease in daytime sleepiness and snoring; however, it has little impact on patient's objective measures of OSA severity including apnea hypoapnea index (AHI).^6^ However, there is no current literature that demonstrates OSA's impact on overall disease severity for CRS patients and furthermore fails to demonstrate how treatment of OSA can impact CRS symptoms. This study aims to identify how a diagnosis of OSA impacts overall CRS disease severity and identify how the treatment of OSA may impact CRS disease severity.

## Methods

This study utilized electronic health care records provided by the TriNetX US Collaborative Network, which encompasses 52 health care organizations and approximately 88 million patients across the United States, from August 22 to 26, 2022. TriNetX, LLC is a global federated health research network that provides a continuously updated database of patient medical statistics. All information is aggregated and deidentified, and as such is exempt from receiving Institutional Review Board (IRB) approval per Thomas Jefferson University's IRB.

This study included data from all patients with a record of a clinical visit after January 1, 2013, the year TriNetx was founded. Cohorts were created based on International Classification of Diseases 10th revision and Current Procedural Terminology (CPT) codes listed in Table 1. Individuals were included that carried 1 or both diagnoses of CRS and/or OSA with a polysomnography. Cohorts were further divided into CRS only, OSA only, and individuals with both OSA and CRS (OSA‐CRS). In order to assess the impact of endotypes, CRS was further divided into chronic rhinosinusitis with nasal polyps (CRSwNP) and chronic rhinosinusitis without nasal polyps (CRSsNP). A diagnosis of CRS (CRS, CRSwNP, and CRSsNP), OSA, or both were considered “index events.” All subsequent analyses required the “index event” to come before any subsequent medical treatment or procedure. Basic demographics and comorbidities including obesity, diabetes mellitus, asthma, nicotine dependence, major depression, and hypertension were collected.

**Table 1:** ICD‐10 Codes for Propensity Score Matching and Patient Outcome CPT Codes

Diagnosis	ICD‐10 code
CRS	J32
CRSwNP	J33, J33.0, J33.8, J33.9
CRSsNP	J32, J32.0‐J32.4, J32.8, J32.9
OSA	G47.33
Asthma	J45
Hypertension	I10‐I16
Nicotine dependence	F17
Anxiety	F40‐48
Major depressive disorder	F33
Obesity	E65‐E68
Diabetes mellitus	E08‐E13

Procedure	CPT code
Functional endoscopic sinus surgery	31253, 31254, 31255, 31256, 31257, 31259, 31267, 31276, 31287, 31288
Tonsillectomy and adenoidectomy	1007178
Uvulopalatopharyngoplasty	42145
Septoplasty (part of exclusion criteria)	30520
Hypoglossal nerve stimulator	64582
Continuous positive airway pressure initiation and management	94660

Abbreviations: CPT, Current Procedural Terminology; CRSsNP, chronic rhinosinusitis without nasal polyps; CRSwNP, chronic rhinosinusitis with nasal polyps; ICD‐10, International Classification of Diseases 10th revision; OSA, obstructive sleep apnea.

To determine the effects of treatment of OSA on CRS, cohorts of untreated individuls or individuals without record of medical or surgical treatment were compared to the treated cohort. The treated cohort was broken down into medical (continuous positive airway pressure [CPAP]) or surgical treatment (tonsillectomy and adenoidectomy, uvulopalatopharyngoplasty [UPPP], and hypoglossal nerve stimulator) (Table 1). This analysis was only done for the total CRS cohort. The CRSwNP and CRSsNP cohorts did not have adequate numbers to run the analysis in TriNetx. Patients who received a septoplasty were excluded. While septoplasty may be used to treat OSA, it has minimal impact on AHI and is likely more important for CPAP compliance. CRS‐related outcomes of interest included rates of endoscopic sinus surgery (ESS), as stated above patients who underwent a septoplasty alone were excluded. CPT codes used for ESS are summarized in Table 1. Oral antibiotic and oral steroid use were also assessed. Oral antibiotics and steroids are both commonly used as adjuvant medical treatment for CRSwNP and CRSsNP.^1^ The oral antibiotics assessed included those commonly associated with treatment of CRS (amoxicillin, amoxicillin/clavulanic acid, trimethoprim, cefpodoxime, cefuroxime, doxycycline, clindamycin, levofloxacin, moxifloxacin, metronidazole, and erythromycin). TriNetX includes specific filters that allow for the inclusion of oral mediations alone.

All statistical analyses were conducted within the TriNetX platform. Propensity score matching for comorbidities (J45, I10‐I16, E08‐E13, F17, F40‐48, Table 1) was conducted for all analyses in order to control for common comorbidities including asthma. For outcomes of interest, risk difference and odds ratios (ORs) with 95% confidence intervals (CIs) were calculated, each with 95% CIs. The Bonferroni method was used to account for multiple hypothesis testing.

## Results

A total of 1,818,879 patients were identified for the CRS only cohort, 481,144 OSA only, and 93,153 patients had CRS‐OSA. The CRS‐OSA cohort was predominantly female (55%) with a mean age of 55.80 ± 20.10 years compared to the CRS (63% female, 52.90 ± 19.10) and OSA (48%, 58 ± 16.60) only cohorts (Table 2). The CRS‐OSA cohort had a greater burden of OSA‐related comorbidities compared to the OSA‐only cohort including hypertension (CRS‐OSA: 63%, OSA: 52%), diabetes (CRS‐OSA: 32%, OSA: 24%), obesity (CRS‐OSA: 58%, OSA: 44%), and cerebral vascular accidents (CRS‐OSA: 13%, OSA: 9%). Comorbidities including asthma (CRS‐OSA: 36%, CRS: 11%), anxiety (CRS‐OSA: 46%, CRS: 16%), nicotine use (CRS‐OSA 20%, CRS 8%), and major depressive disorder (CRS‐OSA: 12%, CRS: 2%) were also higher in the CRS‐OSA cohort when compared to patients with CRS‐only. All results were statistically significant, *P* < 0.0001 (Table 2).

**Table 2:** Cohort Demographics and CRS‐/OSA‐Related Comorbidities

	OSA only (n = 481,144)*	CRS only (n = 1,818,879)*	OSA and CRS (n = 93,153)*
Age (mean years ± SD)	58 ± 16.60	52.90 ± 19.10	55.80 ± 20.10
Sex			
Male	268,722 (52%)	617,554 (37%)	42,046 (45%)
Female	244,590 (48%)	1,071,997 (63%)	51,222 (55%)
Race			
White	346,295 (67%)	1,247,497 (72%)	69,376 (75%)
Black	89,946 (18%)	206,729 (12%)	15,940 (17%)
Asian	8,568 (2%)	29,388 (2%)	764 (1%)
Native American or Pacific Islander	533 (0%)	5,987 (0%)	69 (0%)
Unknown	65,862 (13%)	199,711 (12%)	6801 (7%)
Ethnicity			
Hispanic or Latino	42,519 (8%)	84,948 (5%)	5926 (6%)
Not Hispanic or Latino	383,314 (75%)	1,114,445 (65%)	66,820 (72%)
Unknown	87,522 (17%)	490,780 (30%)	20,524 (22%)
Comorbidities			
Hypertension	171,754 (52%)	108,607 (23%)	56,028 (63%)
Diabetes mellitus	79,292 (24%)	51,960 (11%)	28,657 (32%)
Obesity	147,010 (44%)	46,510 (10%)	51,715 (58%)
Nicotine dependence	44,462 (13%)	39,953 (8%)	18,047 (20%)
Asthma	57,926 (17%)	51,960 (11%)	32,036 (36%)
Anxiety	90,713 (27%)	77,046 (16%)	41,368 (46%)
Major depressive disorder	22,396 (7%)	11,642 (2%)	11,020 (12%)

Abbreviations: CRS, chronic rhinosinusitis; OSA, obstructive sleep apnea.

* All 3 groups were statistically significant at *P* < 0.0001.

After propensity score matching (Table 3), patients in the CRS‐OSA cohort had higher rates of oral antibiotic usage (OR: 1.90, 95% CI: 1.81‐1.96, *P* < 0.001) as well as oral steroid usage (OR: 2.23, 95% CI: 2.16‐2.28, *P* < 0.001). CRS‐OSA also had higher rates of ESS compared to CRS‐only counterparts. 4.51% of patients in the CRS‐OSA cohort compared to 2.54% of CRS‐only patients underwent ESS (OR: 1.91, 95% CI: 1.82‐2.02, *P* < 0.0001) (Table 4).

**Table 3:** CRS‐/OSA‐Related Comorbidities After Propensity Score Matching

	CRS only (n = 91,914)	OSA and CRS (n = 93,153)	*P* value
Hypertension	55,871(60.79%)	55,814 (60.72%)	0.79
Diabetes mellitus	25,910 (28.19%)	26,014 (28.30%)	0.59
Obesity	52,265 (56.86%)	52,265 (56.86%)	1.00
Nicotine dependence	17,818 (19.39%)	17,845 (19.41%)	0.87
Asthma	31,399 (34.16%)	31,341 (34.10%)	0.78
Anxiety	41,246 (44.88%)	41,309 (44.94%)	0.77
Major depressive disorder	10,047 (10.93%)	10,145 (11.04%)	0.47

Abbreviations: CRS, chronic rhinosinusitis; OSA, obstructive sleep apnea.

**Table 4:** Severity of Comorbid CRS and OSA Versus CRS Only Via Antibiotic, Oral Steroid Usage, and ESS

	CRS (n = 1,818,879)	CRS and OSA (93,747)	Odds ratio	95% CI	*P* value
Rate of ESS					
Matched	2191 (2.63%)	4103 (4.93%)	1.91	1.82‐2.02	<0.0001
Unmatched	56,113 (3.34%)	4103 (4.92%)	1.49	1.45‐1.54	<0.0001
Antibiotic use					
Matched	10,403 (21.66%)	15,464 (34.09%)	1.90	1.81‐1.96	<0.0001
Unmatched	233,503 (21.27%)	15,464 (34.09%)	1.92	1.87‐1.95	<0.0001
Oral steroid use					
Matched	9101 (13.00%)	16,691 (24.96%)	2.23	2.16‐2.28	<0.0001
Unmatched	174,110 (11.45%)	16,691 (24.96%)	2.57	2.52‐2.62	<0.0001

Bonferroni corrected *α*, *P* < 0.017 corresponds to *P* < 0.05.

Abbreviations: CI, confidence interval; CRS, chronic rhinosinusitis; ESS, endoscopic sinus surgery; OSA, obstructive sleep apnea.

The CRS cohort was divided in to CRSwNP and CRSsNP. A total of 149,553 patients with CRSwNP and OSA were identified and 346,151 patients with CRSsNP and OSA were included. CRSwNP‐OSA (Table 5) cohort had higher rates of all comorbidities examined compared to the CRS and OSA cohorts alone. After propensity score matching, CRSwNP‐OSA patients had an equivocal disease burden compared to the CRSwNP cohort alone based on rates of ESS and medication use. The CRSwNP‐OSA cohort had higher rates of oral steroid use (OR: 1.12, 95% CI: 1.04‐1.21, *P* = 0.004) while the CRSwNP cohort had higher rates of ESS (OR: 0.69, 95% CI: 0.65‐0.73, *P* < 0.0001). There was no statistical difference in antibiotic use (Table 6). The CRSsNP‐OSA cohort similarly had higher rates of all comorbidities examined compared to OSA and CRS cohorts alone (Table 7). This cohort also had higher rates of ESS (OR: 1.76, 95% CI: 1.70‐1.82, *P* < 0.0001) as well as higher rates of oral steroid use (OR: 1.23, 95% CI: 1.21‐1.26, *P* < 0.0001) than CRSsNP alone. There was no statistical difference in antibiotic use (Table 8).

**Table 5:** Cohort Demographics OSA, CRSwNP, CRSwNP‐OSA‐Related Comorbidities

	OSA only (n = 3,014,263)*	CRSwNP only (n = 149,553)*	CRSwNP and OSA (n = 28,831)*
Age (mean years ± SD)	57.30 ± 20.60	53.9 ± 19.7	59.6 ± 16.9
Sex			
Male	1,664,865 (55%)	72,153 (52%)	17,323 (60%)
Female	1,219,855 (41%)	62,199 (44%)	10,337 (36%)
Race			
White	1,979,539 (66%)	92,739 (67%)	19,821 (68%)
Black	406,309 (13%)	14,183 (10%)	3459 (12%)
Asian	70,509 (2%)	5370 (4%)	710 (3%)
Unknown	423,483 (14%)	20,978 (15%)	3922 (14%)
Ethnicity			
Hispanic or Latino	221,285 (7%)	9153 (7%)	1683 (6%)
Not Hispanic or Latino	2,026,822 (67%)	89,928 (64%)	20,523 (71%)
Unknown	764,614 (26%)	40,508 (29%)	6,759 (23%)
Comorbidities			
Hypertension	200,774 (34.00%)	27,679 (19.92%)	15,499 (53.77%)
Diabetes mellitus	98,380 (17.00%)	10,055 (7.24%)	6880 (23.87%)
Obesity	142,800 (24.00%)	11,689 (8.41%)	11,932 (41.40%)
Nicotine dependence	43,336 (7.00%)	8514 (6.13%)	3869 (13.42%)
Asthma	60,084 (10.00%)	24,675 (17.76%)	10,473 (36.33%)
Anxiety	84,829 (14.00%)	18,186 (13.09%)	9181 (31.85%)
Major depressive disorder	17,546 (3.00%)	2713 (1.95%)	2148 (7.45%)

Abbreviations: CRSwNP, chronic rhinosinusitis with nasal polyps; OSA, obstructive sleep apnea.

* All 3 groups were statistically significant at *P* < 0.0001.

**Table 6:** Severity of Comorbid CRSwNP and OSA Versus CRSwNP Only Via Antibiotic, Oral Steroid Usage, and ESS

	CRSwNP (n = 149,553)	CRSwNP and OSA (n = 28,831)	Odds ratio	95% CI	*P* value
Rate of ESS					
Matched	3782 (15.81%)	2488 (11.48%)	0.69	0.65‐0.73	<0.0001
Unmatched	22,531 (17.84%)	2664 (11.30%)	0.58	0.56‐0.61	<0.0001
Antibiotic use					
Matched	3910 (25.84%)	3360 (24.53%)	0.93	0.88‐0.98	0.010
Unmatched	24,064 (25.11%)	3485 (24.34%)	0.96	0.92‐1.00	0.047
Oral steroid use					
Matched	1287 (5.20%)	1418 (5.80%)	1.12	1.04‐1.21	0.004
Unmatched	5062 (3.77%)	1395 (5.16%)	1.39	1.31‐1.47	<0.0001

Bonferroni corrected *α*, *P* < 0.017 corresponds to *P* < 0.05.

Abbreviations: CI, confidence interval; CRSwNP, chronic rhinosinusitis with nasal polyps; ESS, endoscopic sinus surgery; OSA, obstructive sleep apnea.

**Table 7:** Cohort Demographics CRSsNP, OSA, CRSsNP‐OSA‐Related Comorbidities

	OSA only (n = 3,014,263)*	CRSsNP only (n = 2,187,708)*	CRSsNP and OSA (n = 346,151)*
Age (mean years ± SD)	57.30 ± 20.60	48.4 ± 22.2	58.6 ± 18.30
Sex			
Male	1,664,865 (55%)	752,041 (37%)	162,016 (47%)
Female	1,219,855 (41%)	1,219,209 (60%)	172,429 (49%)
Race			
White	1,979,539 (66%)	1,427,811 (71%)	249,209 (72%)
Black	406,309 (13%)	205,653 (10%)	41,394 (12%)
Asian	70,509 (2%)	48,951 (2%)	5611 (2%)
Unknown	423,483 (14%)	260,653 (13%)	40,832 (12%)
Ethnicity			
Hispanic or Latino	221,285 (7%)	132,424 (7%)	20,042 (6%)
Not Hispanic or Latino	2,026,822 (67%)	1,342,734 (66%)	256,685 (74%)
Unknown	764,614 (26%)	548,537 (27%)	70,734 (20%)
Comorbidities			
Hypertension	200,774 (34.00%)	108,678 (20.00%)	162,895 (57.00%)
Diabetes mellitus	98,380 (17.00%)	41,826 (8.00%)	76,446 (27.00%)
Obesity	142,800 (24.00%)	51,576 (9.00%)	130,029 (45.00%)
Nicotine dependence	43,336 (7.00%)	38,661 (7.00%)	40,803 (14.00%)
Asthma	60,084 (10.00%)	60,255 (11.00%)	83,477 (29.00%)
Anxiety	84,829 (14.00%)	83,520 (15.00%)	109,655 (38.00%)
Major depressive disorder	17,546 (3.00%)	12,455 (2.00%)	25,459 (9.00%)

Abbreviations: CRSsNP, chronic rhinosinusitis without nasal polyps; OSA, obstructive sleep apnea.

* All 3 groups were statistically significant at *P* < 0.0001.

**Table 8:** Severity of Comorbid CRSsNP and OSA Versus CRSsNP Only Via Antibiotic, Oral Steroid Usage, and ESS

	CRSsNP (n = 2,187,708)	CRSsNP and OSA (n = 346,151)	Odds ratio	95% CI	*P* value
Rate of ESS					
Matched	5031 (1.58%)	8513 (2.75%)	1.76	1.70‐1.82	<0.0001
Unmatched	38,278 (1.90%)	9082 (2.70%)	1.43	1.39‐1.47	<0.0001
Antibiotic use					
Matched	42,020 (26.65%)	36,903 (25.54%)	0.94	0.93‐0.96	<0.0001
Unmatched	302,015 (25.44%)	39,031 (25.48%)	1.00	0.99‐1.01	0.73
Oral steroid use					
Matched	18,296 (5.75%)	22,278 (7.00%)	1.23	1.21‐1.26	<0.0001
Unmatched	107,852 (5.33%)	24,611 (7.11%)	1.36	1.34‐1.38	<0.0001

Bonferroni corrected *α*, *P* < 0.017 corresponds to *P* < 0.05.

Abbreviations: CRSsNP, chronic rhinosinusitis without nasal polyps; ESS, endoscopic sinus surgery; OSA, obstructive sleep apnea.

Patients that received both medical and surgical treatment for OSA were then assessed for CRS‐related disease severity. CRS‐OSA patients that used CPAP demonstrated decreased rates of both antibiotic (OR: 3.24, 95% CI: 2.82‐3.71, *P* < 0.0001) and oral steroid use (OR: 2.28, 95% CI: 2.05‐2.55, *P* < 0.0001). However, CRS‐OSA patients on CPAP had equivocal rates of ESS compared to their untreated counterparts. Patients that underwent surgical treatment of OSA demonstrated decreased rates of antibiotic (OR: 1.93, 95% CI: 1.62‐2.28, *P* < 0.0001) consumption. However, they had equivocal rates of oral steroid (OR: 0.95, 95% CI: 0.82‐1.10, *P* = 0.45) consumption compared to OSA‐CRS patients who had not received any of the listed sleep surgeries and slightly higher rates of ESS compared to their untreated counterparts after propensity score matching (Table 9).

**Table 9:** Effects of OSA Medical and Surgical Treatment on CRS Outcomes

Medical (CPAP) treatment	Untreated OSA/CRS (n = 76,362)	Treated OSA/CRS (n = 9,721)	Odds ratio	95% CI	*P* Value
Antibiotic use					
Matched	717 (21.86%)	352 (7.95%)	3.24	2.82‐3.71	<0.0001
Unmatched	6164 (19.75%)	384 (9.53%)	2.34	2.10‐2.61	<0.0001
Oral steroid use					
Matched	1082 (18.76%)	553 (9.18%)	2.28	2.05‐2.55	<0.0001
Unmatched	8373 (15.80%)	726 (11.65%)	1.42	1.31‐1.54	<0.0001
Rates of ESS					
Matched	72 (1.75%)	68 (1.54%)	1.14	0.918‐1.414	0.24
Unmatched	1224 (2.31%)	150 (1.54%)	1.51	1.28‐1.78	<0.001

Surgical treatment	Untreated OSA/CRS (n = 528,910)	Treated OSA/CRS (n = 5211)	Odds ratio	95% CI	*P* value
Antibiotic use					
Matched	516 (16.13%)	230 (9.08%)	1.93	1.62‐2.28	<0.0001
Unmatched	70,721 (17.48%)	230 (12.21%)	1.52	1.32‐1.75	<0.0001
Oral steroid use					
Matched	382 (9.23%)	389 (8.06%)	0.95	0.82‐1.10	0.45
Unmatched	49,139 (9.98%)	389 (9.72%)	1.03	0.92‐1.14	0.63
Rates of ESS					
Matched	63 (1.52%)	198 (2.04%)	0.74	0.59‐0.93	0.009
Unmatched	1015 (1.33%)	198 (2.04%)	0.65	0.55‐0.77	<0.0001

Bonferroni corrected *α*, *P* < 0.017 corresponds to *P* < 0.05.

Abbreviations: CI, confidence interval; CPAP, continuous positive airway pressure; CRS, chronic rhinosinusitis; ESS, endoscopic sinus surgery; OSA, obstructive sleep apnea.

## Discussion

Several studies have described the connection between CRS and OSA, however little is known how comorbid OSA impacts CRS. The current population‐based study investigated CRS severity with relation to a comorbid diagnosis of OSA. Overall, these results demonstrate that patients with CRS‐OSA experience a greater burden of all CRS‐ and OSA‐related comorbidities examined as well as an increase in CRS burden compared to CRS patients alone. Additionally, treatment of OSA reduced both steroid and antibiotic use.

The relationship between OSA and CRS is still being investigated, however the current belief is that apneic events lead to increased production of inflammatory cytokines and contribute to the development of CRS.^7^ This increase in inflammatory cytokines may also contribute to the overall higher rates of related comorbidities within the CRS‐OSA population. The CRS‐OSA population had higher rates of asthma, hypertension, diabetes, and psychiatric diagnoses specifically anxiety and depression than the CRS or OSA populations alone. CRS and OSA are both independently associated with worse outcomes for patients with asthma, therefore, for patients with both CRS and OSA, the burden of both conditions could lead to a possible additive effect.^8^, ^9^ Additionally, the relationship between obesity and OSA has been clearly established, however, recent studies have demonstrated CRS patients have higher rates of obesity.^4^ Therefore, this association with an increased risk of obesity for the CRS‐OSA cohort may also contribute to the higher rates of diabetes and hypertension.

A population‐based study from Taiwan indicated that OSA was a risk factor for the subsequent development of CRS.^4^ The current study's findings align with this hypothesis, and demonstrate that comorbid OSA may lead to overall higher CRS disease burden as CRS‐OSA patients had an increased consumption of both antibiotics and steroids. Additionally, individuals in the CRS‐OSA cohort had higher rates of ESS when compared to the CRS cohort alone.

This association between OSA and its impact on overall CRS disease burden leads to the question if OSA treatment has an impact on CRS outcomes since the reverse has been previously examined. Treatment for OSA is multifactorial. Nasal obstruction has been shown to increase airway resistance and may worsen OSA and OSA symptoms.^10^ After nasal surgery, patients report improved symptoms including less snoring, increased energy levels, increased CPAP compliance, and overall better quality of life. However, AHI does not appear to be impacted by nasal surgery.^6^, ^10^, ^11^ To date, there are no studies investigating whether treating OSA can impact CRS‐related outcomes. The current population‐based study's results suggest that treating OSA medically with CPAP or with sleep‐related surgery such as UPPP or with a hypoglossal nerve stimulator may may have an impact on CRS outcomes for patients with both conditions. Interestingly, this study suggests that treatment with CPAP may be more beneficial for CRS‐related disease. The current study showed that CRS‐OSA patients treated with CPAP demonstrated a greater percentage decrease in antibiotic and steroid consumption when compared to sleep surgery, however, both demonstrated statistically significant differences compared to no treatment.

However, it is important to note that these results shifted when examining the impact of OSA on CRS both with and without nasal polyps. It appears that patients with CRSsNP and OSA are the main driving factor for why CRS‐OSA patients have a higher burden of CRS‐related disease. They had higher rates of both ESS and steroid use. However, those with CRSwNP and OSA had relatively similar rates of surgery, anitibiotic, and steroid use as to patients with CRSwNP alone.

There are important limitations to this study. While the TriNetx database represents a large cohort of patients in the United States, it does not include all medical centers. Therefore, patients who do not receive care at the included institutions are not included in the analysis. Additionally, while the diagnosis of OSA was verified by presence of a polysomnogram, there was insufficient AHI data to further categorize OSA severity for subgroup analyses and due to the limitations of a database, we were not able to assess CPAP compliance. Therefore, while patients may have been prescribed CPAP it was not possible to assess how often patients used their CPAP. Furthermore, we were also unable to assess the effect of each individual OSA surgery on CRS and how the impact of OSA treatment varied depending on whether or not patients had CRS with or without nasal polyps due to insufficient numbers. As the database continues to grow, this would be an important area to re analyze in order to help assess which OSA surgical intervention would be most beneficial for patients who also suffer from CRS. Lastly, due to limitations within the TriNetX database, it was not possible to directly link the antibiotics and steroids to specific encounters. Therefore, it is possible that the antibiotics and steroids prescribed were for conditions other than CRS. However, for this analysis a pre‐existing diagnosis of CRS was required prior to administration of any medications. To further limit this bias, only oral antibiotics associated with treatment of CRS exacerbations were included.

## Conclusion

Patients with CRS and comorbid OSA demonstrate increased rates of both OSA‐ and CRS‐related disease sequelae and CRS disease burden. Howevever, OSA may have a larger impact on patients with CRSsNP than those with CRSwNP. Patients who received both medical and surgical management of their OSA appeared to have an improvement in their CRS through decreased steroid and antibiotic use. This is the first study to suggest an improvement in CRS‐related disease after medical or surgical treatment of OSA. This possible improvement is likely secondary to decreased apneic events and subsequent reduction in inflammation linking the 2 conditions.

## Author Contributions

**Emily Garvey**, design, data collection, data analysis, manuscript writing, manuscript preparation; **Alexander Duffy**, data collection, data analysis, manuscript writing, manuscript preparation; **Sruti Tekumalla**, data collection, data analysis, manuscript writing, manuscript preparation; **Bita Naimi**, data collection, data analysis, manuscript preparation; **Chase Kahn**, manuscript preparation; **Angela Yang**, design, manuscript preparation; **Zachary Urdang**, data analysis, manuscript preparation; **Douglas Farquhar**, manuscript preparation; **Marc Rosen**, manuscript preparation; **Gurston G. Nyquist**, design, manuscript preparation; **Elina Toskala**, design, manuscript preparation; **Mindy Rabinowitz**, manuscript preparation.

## Disclosures

### Competing interests

None.

### Funding source

None.
